# Association between serum ferritin level and the various stages of non-alcoholic fatty liver disease: A systematic review

**DOI:** 10.3389/fmed.2022.934989

**Published:** 2022-08-03

**Authors:** Huanqiu Wang, Ruyu Sun, Sisi Yang, Xueqing Ma, Chengbo Yu

**Affiliations:** ^1^State Key Laboratory for Diagnosis and Treatment of Infectious Diseases, National Clinical Research Center for Infectious Diseases, Collaborative Innovation Center for Diagnosis and Treatment of Infectious Diseases, The First Affiliated Hospital, School of Medicine, Zhejiang University, Hangzhou, China; ^2^Institute of Social Medicine, School of Medicine, Zhejiang University, Hangzhou, China

**Keywords:** non-invasive predictor, clinical evaluation, hepatic fibrosis, hepatic steatosis, hepatic inflammation, non-alcoholic steatohepatitis

## Abstract

**Introduction:**

Non-alcoholic fatty liver disease (NAFLD) has become the most common liver disorder across the world, and non-invasive evaluation approaches are in need to assess NAFLD disease progression. Serum ferritin has been proposed as one of the biomarkers for NAFLD diagnosis in previous studies. This systematic review aims to identify, report, and synthesize studies that investigated the association of serum ferritin level with the various stages of NAFLD among the adult population.

**Methods:**

Three databases – MEDLINE, EMBASE, and Scopus – were systematically searched to obtain potentially relevant publications before July 2022. No restrictions were applied to geographical region, study design, publication type and language. The association between serum ferritin level or different ferritin categories and the various stages of NAFLD was the primary outcome of interest. Title and abstract screenings, data extraction and coding, and quality assessment were independently completed by two authors with discrepancies resolved through discussion with a third author.

**Results:**

Thirty-two studies were included and heterogeneity was considerable. The associations between serum ferritin level and the stages of hepatic steatosis, fibrosis, inflammation and ballooning and the occurrence of non-alcoholic steatohepatitis (NASH) were investigated but inconsistent associations were reported. Most studies identified serum ferritin to be a predictor of advanced NAFLD, while several revealed the opposite end.

**Conclusions:**

Serum ferritin could be considered to act as a non-invasive biomarker for assessing various stages of NAFLD. Nevertheless, further studies are still in need to confirm its predictive value since this study reported inconsistent associations based on the qualitative synthesis.

**Systematic Review Registration:**

http://www.crd.york.ac.uk/PROSPERO, identifier: CRD42021275630.

## Introduction

Non-alcoholic fatty liver disease (NAFLD) represents a spectrum of hepatic pathology with fat excessively accumulating in the hepatic parenchyma in individuals who consume little or no alcohol (1, 2). It has become the most common liver disorder across the world, with a global prevalence estimated to be 25.24% (3) and still on the rise (4), heavy in both clinical and economic burdens. Noticeably, sex differences in NAFLD exist – NAFLD is more prevalent and more severe in men than in women during the reproductive age; the differences usually get smaller after menopause (5).

**Figure 1:**
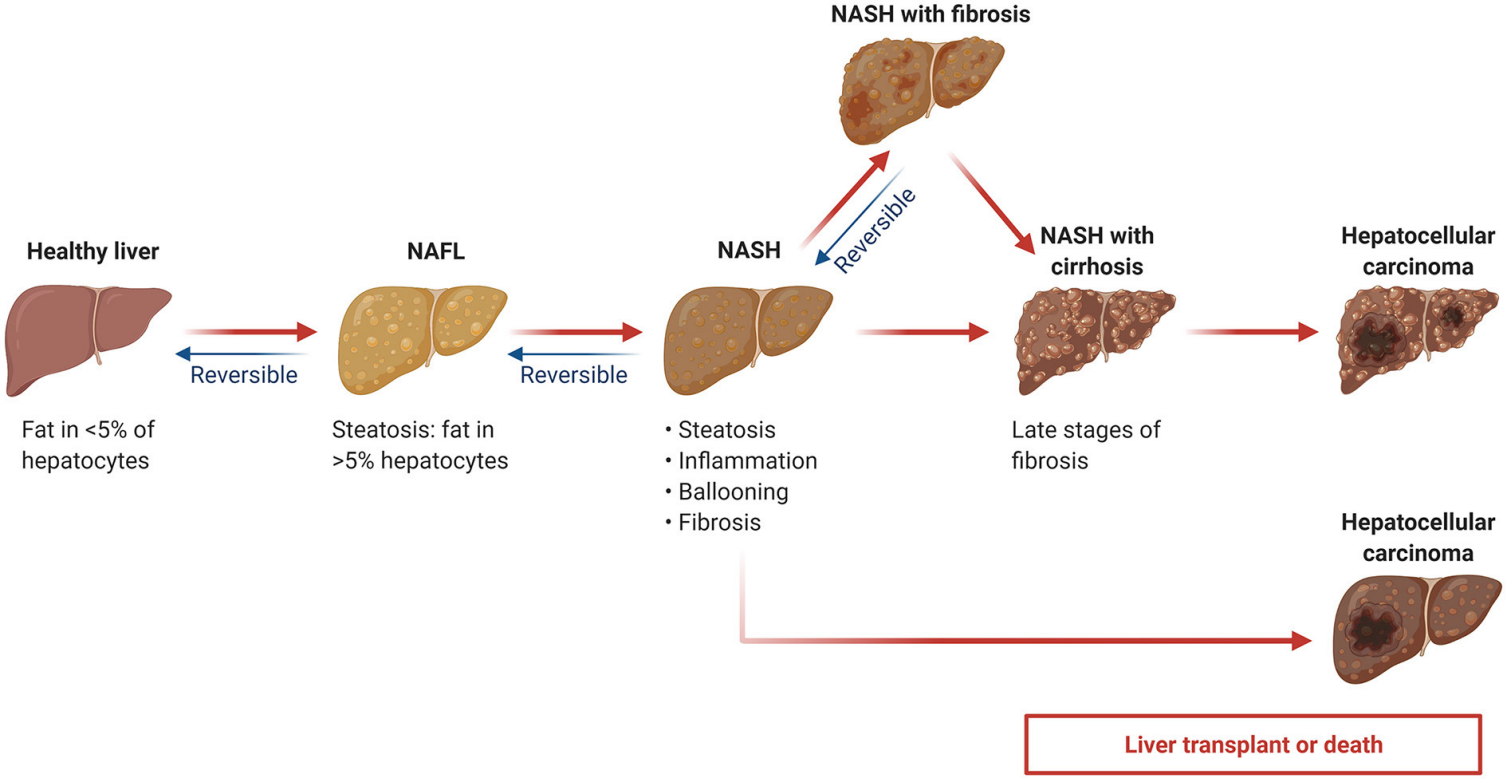
Non-alcoholic fatty liver disease (NAFLD) progression. NAFL: non-alcoholic fatty liver; NASH: non-alcoholic steatohepatitis. Adapted from “Non-Alcoholic Fatty Liver Disease (NAFLD) Spectrum”, by BioRender.com (2022). Retrieved from https://app.biorender.com/biorender-templates.

Generally, NAFLD consists of two subtypes: the first is simple steatosis (also termed as NAFL), which is nonprogressive; the second is non-alcoholic steatohepatitis (NASH), which has not only steatosis but also hepatocyte damage (6). NASH is progressive and may lead to end-stage liver diseases such as fibrosis, cirrhosis, and hepatocellular carcinoma, possibly resulting in liver-related mortality (Figure 1) (7, 8). In the United States, one of the major causes of adult cirrhosis is NASH, with NASH-related cirrhosis recognized as the second indication for liver transplantation (3). Hence, clinical evaluation of the disease progression in NAFLD patients is important for physicians to choose appropriate interventions and assess prognosis.

According to current clinical guidelines, liver biopsy is heavily relied upon for the clinical evaluation of NAFLD, especially for the diagnosis of NASH (9). However, liver biopsy is an invasive procedure and may be accompanied by complications such as bleeding (10), and there might be underestimation of the disease progression, which is caused by sampling bias, since a biopsy specimen represents only ~1/50,000 of the liver volume (11). Therefore, it is suggested to develop and utilize accurate non-invasive evaluation approaches such as imaging and biomarkers, either to combine with liver biopsy for higher validity and reliability, or replace it to avoid invasive diagnostic procedures.

Serum ferritin has been widely studied to assist with disease diagnosis and progression, since it is an acute-phase reactant and a pro-inflammatory cytokine whose concentration is elevated in both infectious and non-infectious inflammation (12). Elevated serum ferritin is reported in about 30% patients diagnosed with NAFLD (13) and it has been proposed as one of the biomarkers for NAFLD diagnosis in previous studies (11, 14). For instance, one Iranian study proposed that the ferritin values of 150 ng/ml in females and 248 ng/ml in males as potential diagnostic cut-off points (15). Studies have also identified it as a potential indicator for the evaluation of NAFLD progression and prognosis, e.g., predicting liver fibrosis in NAFLD patients (16).

To the best of our knowledge, few studies have synthesized existing evidence on the association between serum ferritin and disease progression of NAFLD. This systematic review aims to address the research gap by identifying, reporting, and synthesizing studies that investigated the association of serum ferritin level or different ferritin categories with the various stages of NAFLD among the adult population.

## Methods

This study was conducted following the Preferred Reporting Items for Systematic Reviews and Meta-Analysis (PRISMA) guidelines (17), and was prospectively registered with PROSPERO (protocol number CRD42021275630; http://www.crd.york.ac.uk/PROSPERO).

### Search strategy and eligibility criteria

Three databases – MEDLINE, EMBASE, and Scopus – were systematically searched using a combination of the key terms “ferritin,” “fatty liver,” “hepatic steatosis,” “non-alcoholic steatohepatitis” and related syntax (title/abstract/keywords/MeSH) to obtain potentially relevant publications before July 2022. No restrictions were applied to geographical region, study design, publication type and language. Full search strategies are presented in Supplementary Table 1.

The inclusion criteria were as follows: (1) original and empirical human studies; (2) observational studies including cross-sectional, case-control, and cohort studies; (3) studies that enrolled adult NAFLD patients diagnosed with any approach; (4) studies that explored the association between serum ferritin and disease progression of NAFLD, with confounding factors either adjusted or not.

The exclusion criteria were as follows: (1) review, case-report, abstract, protocol, letter, commentary, meta-analysis and proceeding articles; (2) interventional studies such as clinical trials; (3) experiments performed *in vitro* or in animals; (4) studies that included pediatric patients or patients diagnosed with other chronic liver diseases, e.g., hepatitis B and C, autoimmune hepatitis, etc.; (5) studies not analyzing the association between serum ferritin and disease progression of NAFLD.

Results identified from the search were imported into a citation manager (Zotero), and duplicates were removed. Two authors (HW and RS) independently screened titles and abstracts against the eligibility criteria. Full texts were retrieved for evaluation when citations were considered relevant or with insufficient information for inclusion or exclusion during title/abstract screening. Manual searches were conducted in the reference lists of included studies to obtain additional relevant studies. Full-text evaluations were independently conducted by two authors (HW and RS). Disagreements between the two authors during screening and evaluation were discussed with a third author (CY) to reach consensus.

### Data extraction and quality assessment

Data was extracted from the included studies using a purposive-built data collection form in Excel. The following data was extracted and coded into the form: (1) publication information including first author's name, article title, year of publication; (2) study design including study type, study location, sample size, target population, and selection criteria for participant recruitment; (3) socio-demographic status and medical history of study participants; (4) results of liver imageology (ultrasound, CT, or MRT) and liver biopsy, including grades of steatosis, ballooning, inflammation, fibrosis, cirrhosis, etc.; (5) serum ferritin level, together with its testing methods; (6) approaches or standards employed for NAFLD diagnosis and grading; (7) proven associations between serum ferritin level and the various stages of NAFLD. Two authors (HW and RS) independently extracted and coded the data. Discrepancies during this process was discussed with a third author (CY) until consensus was reached.

Quality of the included studies was assessed using the quality control criteria for proteomic studies reporting potential biomarkers (18). Quality assessment was independently completed by two authors (HW and RS), and disagreements were resolved through discussion with a third author (CY).

## Results

**Figure 2:**
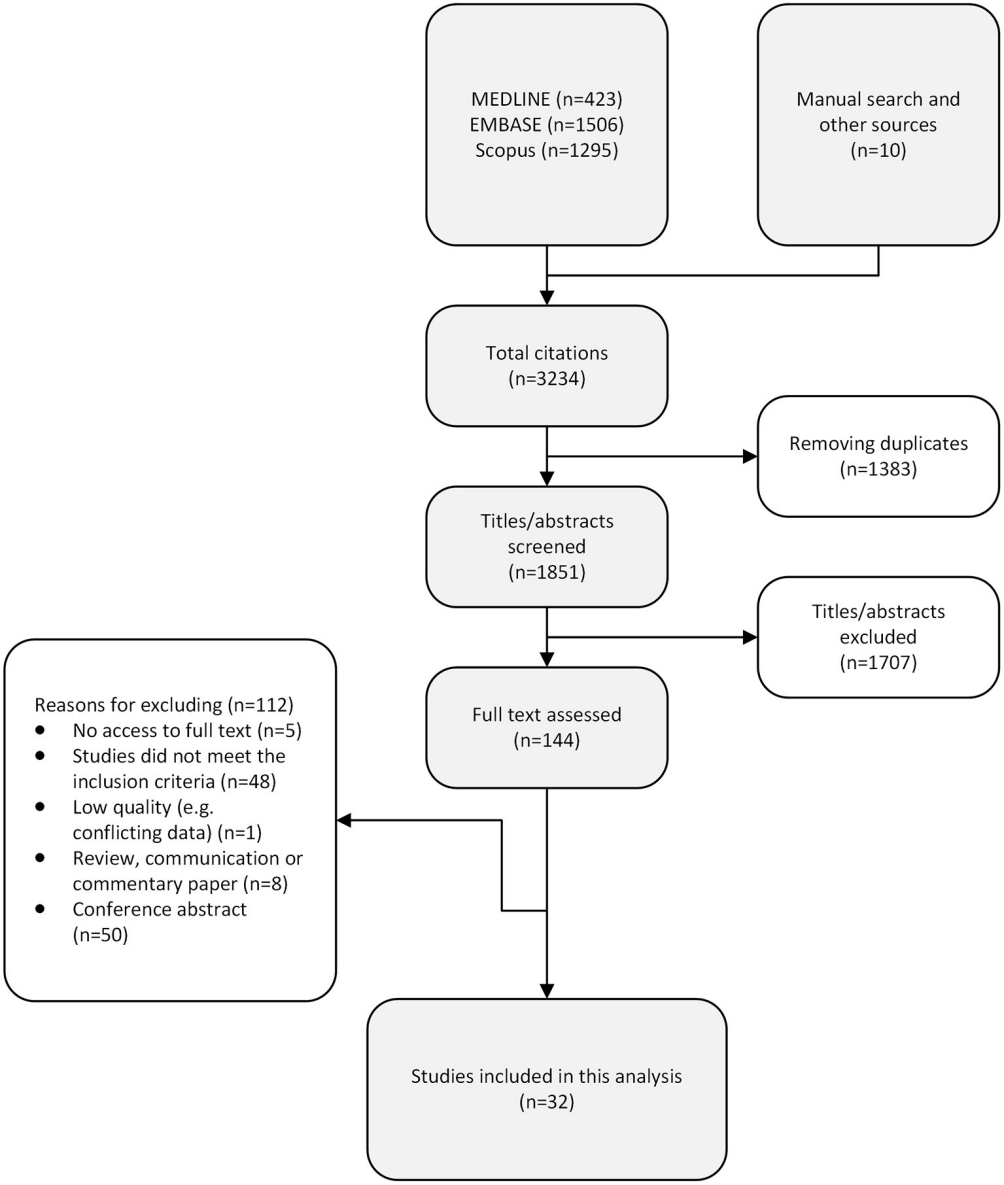
Flowchart of study identification and selection.

**Figure 3:**
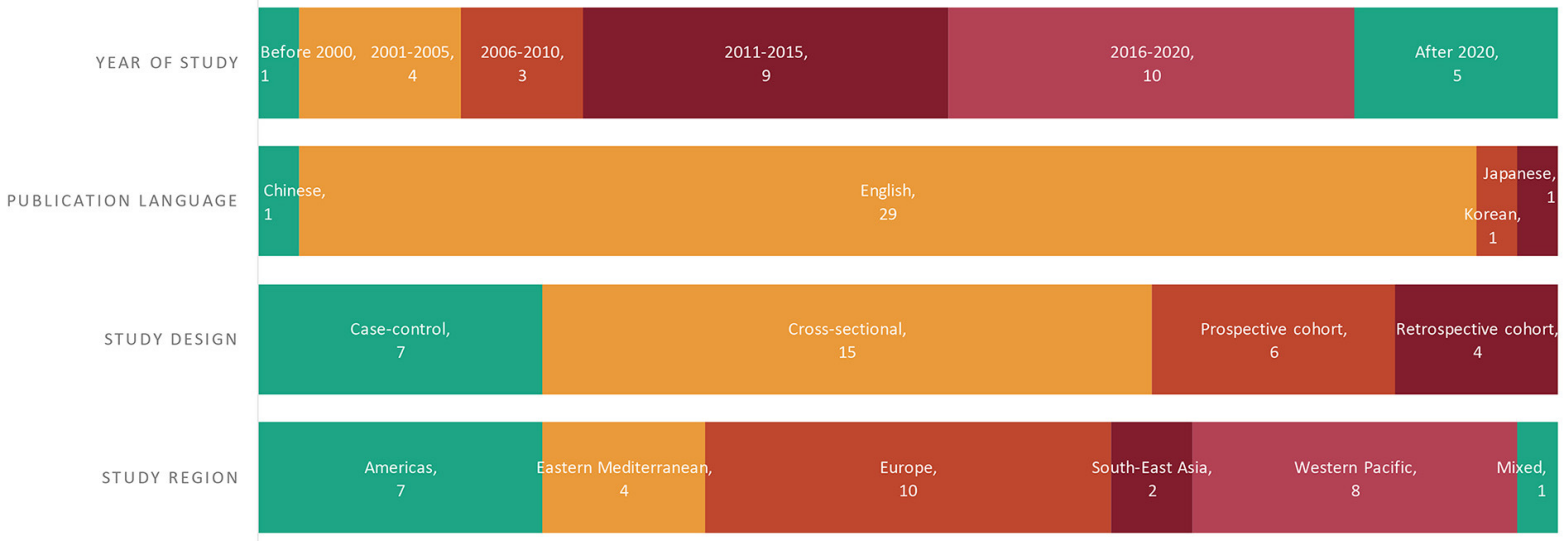
Summary of characteristics of included studies.

A total of 3,234 records were returned from the literature search, of which 1,383 duplicates were removed and 1,707 citations were excluded during title/abstract screenings (Figure 2). We assessed 144 full-text articles, and 32 studies met the predefined inclusion criteria. Figure 3 presents the characteristics of all included studies, categorized by year of study, publication language, study design, study region [the World Health Organization (WHO) regions], and participants. Nearly half of the included studies were published after 2016 (*n* = 15, 46.9%; Figure 3). Most of the studies were published in English (*n* = 29, 90.6%), with another one study published in Chinese, one study published in Japanese, and one study published in Korean. Fifteen studies employed cross-sectional design, 10 studies were cohort studies, and seven studies were case-control studies. The included studies covered a total of 28,261 participants, of whom 27,028 were NAFLD patients, including 2,376 NASH patients; one study explored the association of ferritin and the various stages of NAFLD in patients with hypothyroidism (Table 1).

**Table 1:** Characteristics and findings of included studies.

**Study (first author, year)**	**Study design**	**Country**	**Participants**	**Sample size (M + F)**	**Mean age ± SD (yrs)**	**NAFLD diagnosis approach/tool**	**NAFLD progression approach/tool**	**Number of NAFLD patients with different gradings, n (%)**	**Serum ferritin level, mean ± SD (ng/ml)**	**Proven associations by univariate analysis, OR (95% CI) and *p*-value were reported in logistic regression analysis and correlation coefficient (*r*) and *p*-value were reported in correlation analysis**	**Proven associations by multivariate analysis, OR (95% CI) and *p*-value were reported in logistic regression analysis and correlation coefficient (*r*) and *p*-value were reported in correlation analysis**	**Confounders adjusted for**
Angulo, 2014 (19)	Retrospective cohort study	UK, Australia, Italy, US	Adult NAFLD patients	1,014 (586 + 428)	46.9 ± 0.4	Liver biopsy	Liver biopsy Presence of NASH was recorded on pattern and distribution of liver histologic lesions The stage of fibrosis [Kleiner et al. (20)]: stage 0 = absence of fibrosis stage 1 = perisinusoidal or portal stage 2 = perisinusoidal and portal/periportal stage 3 = septal or bridging fibrosis stage 4 = cirrhosis Advanced fibrosis: stage 3–4 fibrosis	NASH category: No NASH: 495 Suspicious/borderline: 129 Definitive: 390 Fibrosis stage: Stage 0: 351 Stage 1: 251 Stage 2: 141 Stage 3: 161 Stage 4: 110	252 ± 8 Normal ferritin: 683 patients Elevated ferritin level: 331 patients ULN: 200 ng/ml in females and 300 ng/ml in males	χ^2^ test: Ferritin and NASH category: *p* = 0.003 Ferritin and fibrosis stage: *p* = 0.003 in women, *p* < 0.001 in men	Multivariate logistic regressions: Presence of fibrosis (stage 1–4): Ferritin > ULN vs. ferritin ≤ ULN: 1.84 (1.36, 2.50), *p* < 0.001 Ferritin > 1.5 ULN vs. ferritin ≤ 1.5 ULN: 2.14 (1.45, 3.15), *p* < 0.001 Ferritin ≤ 2 ULN vs. ferritin > 2 ULN: 2.52 (1.45, 4.41), *p* = 0.001 Severe fibrosis (stage 2–4): Ferritin > ULN vs. ferritin ≤ ULN: 1.64 (1.22, 2.19), *p* = 0.001 Ferritin > 1.5 ULN vs. ferritin ≤ 1.5 ULN: 1.95 (1.38, 2.75), *p* < 0.001 Ferritin ≤ 2 ULN vs. ferritin > 2 ULN: 2.02 (1.30, 3.14), *p* = 0.002 Advanced fibrosis (stage 3 or 4) Ferritin > ULN vs. ferritin ≤ ULN: 1.61 (1.17, 2.18), *p* = 0.004 Ferritin > 1.5 ULN vs. ferritin ≤ 1.5 ULN: 1.95 (1.34, 2.82), *p* < 0.001 Ferritin ≤ 2 ULN vs. ferritin > 2 ULN: 2.11 (1.33, 3.34), *p* = 0.001	Age, sex, race, BMI, diabetes, ALT, and recruitment site
Buzzetti, 2019 (21)	Retrospective cohort study	UK, Italy	Adult NAFLD patients	468 (291 + 177)	47 ± 13	Liver biopsy	Liver biopsy NAFLD lesions were scored according to the NASH Clinical Research Network (CRN) NAS scoring system Hepatic fibrosis [Brunt et al. (22)]: 0 = absence of fibrosis 1 = zone 3 perisinusoidal/perivenular fibrosis 2 = zone 3 and periportal fibrosis 3 = septal/bridging fibrosis 4 = cirrhosis Significant fibrosis: stages ≥F2 Advanced fibrosis: stages ≥F3	No NASH: 221 (47) NASH: 247 (53) F0: 207 (44) F1: 104 (22) F2: 68 (15) F3: 41 (9) F4: 48 (10) ≥F3: 89 (19)	188 (range 61-314) Ferritin > ULN: 122 (26%) patients ULN: 200 ng/ml in females and 300 ng/ml in male	Ferritin and the occurrence of NASH: *p* = 0.42 (*t*-test) Ferritin > ULN and the occurrence of NASH: *p* = 0.11 (χ^2^ test) Ferritin and the occurrence of NASH: 1.001 (1.000-1.001), *p* = 0.07 (univariate logistic regression analysis) Ferritin and fibrosis stage (*t*-test): F0-F1 vs. F2: *p* > 0.05 F0-F1 vs. F3: *p* = 0.024 F0–F1 vs. F4: *p* = 0.028 F2 vs. F3: *p* > 0.05	Multivariate logistic regression analysis: Ferritin and the occurrence of NASH: *p* > 0.05 Ferritin and advanced fibrosis: *p* > 0.05	Ferritin and the occurrence of NASH: Age, sex, BMI, hypertension, diabetes, ALT, iron pattern; Ferritin and advanced fibrosis: not reported
Canbakan, 2007 (23)	Prospective cohort study	Turkey	Adult NAFLD patients	105 (54 + 51)	46.6 ± 9.7	Liver biopsy	Liver biopsy Steatosis and fibrosis were staged according to Brunt et al. NAFL: hepatocellular steatosis without fibrosis and prominent inflammation; NASH: steatosis, ballooning degeneration of hepatocytes, mixed acute and chronic lobular inflammation, and zone 3 perisinusoidal fibrosis	NAFL: 38 NASH: 67 Advanced fibrosis (stage 3): 7 Cirrhosis: 4	NAFL group: 71.2 ± 58.2 NASH group: 109.7 ± 81.5	Ferritin and the occurrence of NASH: *p* = 0.016 (*t*-test) Ferritin and fibrosis stage: *r* = 0.35, *p* < 0.001 (correlation analysis)	N/A	N/A
Chandok, 2012 (24)	Prospective cohort study	Canada	Adult NAFLD patients	482 (227 + 255)	49.6 ± 3.1	Liver biopsy or imaging (either liver ultrasound, CT, or MRI)	Liver biopsy or imaging Patients were stratified into three groups based on their histologic severity of disease and radiologic findings: simple steatosis, NASH or cirrhosis.	NAFLD without cirrhosis or biopsy: 374 Non-NASH: 60 NASH: 28 Cirrhosis: 20	199.6 Serum ferritin ≥ 500: 26 patients Simple steatosis group: 223.9 ±204.6 NASH group: 240.7 ± 228.5 Cirrhosis group: 271.3 ± 536.8	ANOVA test: Ferritin among different groups: *p* = 0.34	N/A	N/A
Chaturvedi, 2020 (25)	Cross-sectional study	India	Adult patients with hypothyroidism, including NAFLD patients	100 (33 + 67) NAFLD patients: 33 (13 + 20)	Not reported	Liver ultrasound	Liver ultrasound Steatosis was graded according to Brunt et al. (22)	Fatty liver grade I: 15 Fatty liver grade II: 12 Fatty liver grade III: 6	Male: Grade I NAFLD: 163.60 ± 148.28 Grade II NAFLD: 527.33 ± 144.20 Grade III NAFLD: 590.50 ± 110.59 Female: Grade I NAFLD: 171.68 ± 202.00 Grade II NAFLD: 241.71 ± 105.67 Grade III NAFLD: 364 (only one patient)	Correlation analysis: Ferritin and steatosis stage among males: *r* = 10.076, *p* = 0.004 Ferritin and steatosis stage among females: *r* = 0.876, *p* = 0.043	N/A	N/A
El Nakeeb, 2017 (26)	Case-control study	Egypt	Group 1: Healthy adults (control group) Group 2: adult NAFLD patients without hepatic fibrosis Group 3: adult NAFLD patients with hepatic fibrosis	113 (Sex not reported) Group 1: 30 Group 2: 31 Group 3: 52	Group 1: 28.03 ± 6.99 Group 2: 29.94 ± 9.27 Group 3: 32.92 ± 12.66	Liver biopsy	Liver biopsy Steatosis and fibrosis were graded according to Brunt et al. (22)	Steatosis (among Group 2&3): S1: 65 S2: 18 S3–S4: 0 Fibrosis (among Group 3): F1: 37 F2: 5 F3: 10	Group 1: 51.95 ± 39.38 Group 2: 76.94 ± 57.41 Group 3: 114.55 ± 120.85	Correlation analysis Ferritin and steatosis stage in Group 2: *r* = 0.009, *p* = 0.962 Ferritin and steatosis stage in Group 3: *r* = 0.745, *p* < 0.001 Univariate logistic regression analysis: Ferritin and the occurrence of fibrosis: *p* = 0.330	N/A	N/A
Fracanzani, 2011 (27)	Cross-sectional study	Italy	Adult NAFLD patients	431 (360 + 71)	Not reported	Liver biopsy	Liver biopsy Steatosis and fibrosis were graded according to Kleiner et al. (20)	No NASH: 174 NASH: 257 Steatosis: grade 1: 219 grade 2: 140 grade 3: 70 Fibrosis 0–1: 301 Fibrosis 2: 130	Ferritin <160 ng/ml: 132 patients Ferritin 161–380 ng/ml: 136 patients Ferritin >380 ng/ml: 131 patients Ferritin was categorized according to tertiles	Univariate logistic regression analysis: Ferritin and the occurrence of NASH: Ferritin <160 ng/ml: Ref Ferritin 161–380 ng/ml: 1.14 (0.7–1.87) Ferritin >380 ng/ml: 1.86 (1.11–3.10) *p* = 0.018 Ferritin and fibrosis stage: Ferritin <160 ng/ml: Ref Ferritin 161–380 ng/ml: 1.42 (0.83–2.43) Ferritin >380 ng/ml: 1.40 (0.82–2.40) *p* = 0.21	Multivariate logistic regression analysis: Ferritin and the occurrence of NASH: Ferritin <160 ng/ml: Ref Ferritin 161–380 ng/ml: 1.04 (0.55–1.98) Ferritin >380 ng/ml: 2.06 (0.98–4.33) *p* = 0.07 Ferritin and fibrosis stage: Ferritin <160 ng/ml: Ref Ferritin 161–380 ng/ml: 1.61 (0.79–3.31) Ferritin >380 ng/ml: 3.39 (1.42–8.12) *p* = 0.006	Referral center, gender, age, waist circumference, ALT, HOMA-IR, glucose tolerance, metabolic syndrome, steatosis stage or fibrosis stage
Goh, 2016 (28)	Prospective cohort study	US	Adult NAFLD patients	405 (179 + 226)	48 ± 12	Liver biopsy	Liver biopsy Stages of fibrosis, ballooning, steatosis and inflammation were diagnosed following the classification of Kleiner et al. (20) The degree of steatosis (0–3), lobular inflammation (0–3), and ballooning (0–2), made up the 8-point NAFLD activity score (NAS)	NAFL group: 114 (28) NASH group: 291 (72)	NAFL group: 125.7 [61.0, 243.5] NASH group: 184.0 [91.6, 383.0]	ANOVA test: Ferritin and the occurrence of NASH: *p* < 0.001 Correlation analysis: ρ (95% CI) Ferritin and fibrosis stage: 0.09 (−0.01, 0.18), *p* = 0.088 Ferritin and ballooning stage: 0.12 (0.02, 0.21), *p* = 0.021 Ferritin and steatosis stage: 0.16 (0.06, 0.26), *p* = 0.001	N/A	N/A
										Ferritin and inflammation stage: 0.06 (−0.04, 0.16), *p* = 0.24 Ferritin and NAS: 0.15 (0.05, 0.25), *p* = 0.003		
Hagstrom, 2016 (29)	Prospective cohort study	Sweden	Adult NAFLD patients	222 (134 + 88)	Not reported	Liver biopsy	Liver biopsy The degree of steatosis (0–3), lobular inflammation (0–3), and ballooning (0–2), made up the 8-point NAFLD activity score (NAS) Portal inflammation was scored on a scale of 0–4. Fibrosis was scored according to METAVIR (0–4)	Steatosis grade 1: 73 2: 64 3: 51 Lobular inflammation: 0: 13 1: 78 2: 99 3: 32 Ballooning: 0: 61 1: 78 2: 83 Fibrosis stage: 0: 45 1:85 2: 63 3: 20 4: 9	Normal ferritin level: 133 patients High ferritin level: 89 patients Cut-offs: 150 ng/ml in females and 350 ng/ml in males	Fisher's exact test: Ferritin (normal/high) and steatosis grade: *p* = 0.006 Ferritin (normal/high) and lobular inflammation: *p* = 0.004 Ferritin (normal/high) and ballooning: *p* = 0.002 Ferritin (normal/high) and fibrosis stage: *p* < 0.001 Ferritin and NAS: *p* < 0.001 Poisson regression: Following 15 years after biopsy, the high ferritin group showed a significant and gradually steeper increase in mortality. Thirty years after biopsy, the hazard was over 0.10 deaths per person-year in the high ferritin group and <0.03 deaths per person-year in the normal ferritin group. The hazard ratio increased 9% faster each year in the high ferritin group (HR = 1.09, 95% CI: 1.01, 1.18, *p* < 0.05)	After adjusting for potential confounders, the hazard ratio remained essentially unchanged (HR = 1.10, 95% CI 1.01–1.21, *p* < 0.05)	Age at baseline (time for liver biopsy), time, the interaction between time and serum ferritin, sex, smoking, diabetes mellitus type 2, hypertension, BMI and fibrosis stage
Hanafy, 2019 (30)	Case-control study	Egypt	Group 1: healthy adults (control group) Group 2: adult NAFLD patients Group 3: adult NAFLD patients (validation group)	433 (299 + 134) Group 1: 100 (70 + 30) Group 2: 272 (190 + 82) Group 3: 61 (39 + 22)	Group 1: 38.2 ± 1.8 Group 2: 35.5 ± 4.7 Group 3: 40 ± 4.5	Liver ultrasound	Liver ultrasound and biopsy The degree of steatosis was measured by controlled attenuation parameter (CAP) *via* ultrasound: S0: 212–265 dB/m; S1 (5–33% steatosis): 266–303 dB/m; S2 (34–66%): 304–320 dB/m; S3(>66%): 321–400 dB/m	Steatosis: S0: 71 S1: 21 S2: 77 S3: 103 Fibrosis: F0: 66 F1: 26 F2: 26	Group 1: 196 ± 18.7 Group 2: 383 ± 40.2 Group 3: 402 ± 20.2	Correlation analysis: Ferritin and steatosis grade: *r* = 0.664, *p* < 0.001	Logistic regressive analysis: Ferritin and the presence of significant fibrosis: *p* < 0.001	Age and sex
							Fibrosis was measured *via* liver biopsy: significant fibrosis is defined as stage 3, cirrhosis as stage 4	F3: 82 F4: 72				
Kawanaka, 2012 (31)	Prospective cohort study	Japan	Adult NASH patients with stage 3 fibrosis	33 (14 + 19)	57.4 ± 14.4	Liver biopsy	Liver biopsy NASH was diagnosed according to Matteoni et al. (8). Fibrosis was staged according to Brunt et al. (22)	Baseline: F3: 33 Follow up: F0: 0 F1: 9 (improved) F2: 11 (improved) F3: 7 (not changed) F4 (cirrhosis): 6 (deteriorated)	Changes in ferritin between follow-up and baseline: Deterioration group (F4): + 93 No-change group (F3): −109 Improvement group (F1/2): −207	Wilcox rank sum test: Changes in ferritin between follow-up and baseline among three groups: *p* = 0.009 Difference of ferritin changes between no-change group and deterioration group: *p* < 0.05 Difference of ferritin changes between no-change group and improvement group: *p* < 0.05	N/A	N/A
Kim, 2013 (32)	Prospective cohort study	South Korea	Adult NAFLD patients	108 (73 + 35)	39.0 ± 13.5	Liver biopsy	Liver biopsy Histological grading and staging of NAFLD were scored semi-quantitatively according to the original criteria for NAFLD subtypes, and NAFLD histologic activity score (NAS) system. The NAS identified the degree of steatosis (0–3), lobular inflammation (0–3), and hepatocellular ballooning (0–2). The NAS was the sum of above numerical pathologic scores and ranged from 0 to 8 NAFLD type 1: steatosis alone; NAFLD type 2: steatosis with lobular inflammation only; NAFLD type 3: steatosis with hepatocellular ballooning; NAFLD type 4: steatosis with Mallory-Denk bodies or fibrosis NAFLD subtypes 3 and 4 were considered to represent NASH	NAFLD type 1: 1 (1) NAFLD type 2: 40 (37.0) NAFLD type 3: 39 (36.1) NAFLD type 4: 28 (25.9) NASH: 67 (62.0) NAS ≤ 2: 9 (8.3) NAS 3–4: 54 (50) NAS≥5: 45 (41.7) Fibrosis stage: F0: 19 F1: 54 F2: 27 F3: 10 F4: 1	Not reported	Correlation analysis: Ferritin and steatosis stage: *r* = 0.162, *p* = 0.153 Ferritin and inflammation stage: *r* = 0.172, *p* = 0.129 Ferritin and ballooning stage: *r* = 0.127, *p* = 0.266 Ferritin and fibrosis stage: *r* = 0.272, *p* = 0.015 Ferritin and NAS score: *r* = 0.258, *p* = 0.022 Ferritin and NAFLD subtype: *r* = 0.195, *p* = 0.085	N/A	N/A
							Histologic finding with stage 2 or above fibrosis were also defined as NASH The stage of fibrosis was scored according to Brunt et al. (22)					
Kowdley, 2012 (33)	Cross-sectional study	US	Adult NAFLD patients	628 (235 + 393)	47.7 ± 11.8	Liver biopsy	Liver biopsy Histologic features of NAFLD were assessed by the Pathology Committee of the NASH Clinical Research Network in a centralized consensus review format	Steatosis: S1: 252 S2: 214 S3: 162 Lobular inflammation: <2 under 20x: 319 2–4 under 20x: 238 >4 under 20x: 71 Hepatocellular ballooning None: 199 Mild: 161 More than mild: 268 Fibrosis stage: F0: 160 F1: 182 F2: 121 F3: 112 Cirrhosis: 49 NASH diagnosis category: No NASH: 125 Suspicious/borderline: 119 Definite: 384	Ferritin ≤ ULN: 416 patients Ferritin >ULN and ≤ 1.5 × ULN: 84 patients Ferritin >1.5 × ULN: 128 patients ULN: 200 ng/ml in females and 300 ng/ml in males	χ^2^ test: Ferritin and steatosis grade: *p* <0 .001 Ferritin and lobular inflammation: *p* = 0.026 Ferritin and hepatocellular ballooning: *p* = 0.004 Ferritin and fibrosis stage: *p* < 0.001 Ferritin and NASH diagnosis category: *p* = 0.013	Multivariate logistic regression analysis: Ferritin >1.5 × ULN and the presence of advanced fibrosis (i.e., stage 3 or 4): OR = 1.67; *p* = 0.028 Ferritin >2.5 × ULN and the presence of advanced fibrosis: OR = 2.46; *p* = 0.005	Age at biopsy, sex, presence of diabetes, BMI, ALT
Loguercio, 2004 (34)	Cross-sectional study	Italy	Adult NAFLD patients	305 (250 + 55)	Not reported	Liver biopsy	Liver biopsy Presence/absence and the entity of steatosis, intra-acinar and portal inflammation, pericellular, perivenular and portal fibrosis, and cirrhosis were semi-quantitatively assessed as follows: absent = 0; mild ≤ 25%; moderate = 25–75%; severe ≥ 75%	Steatosis: Absent: 0 Mild: 31.5% Moderate: 49.5% Severe: 18.4% Portal fibrosis: Absent: 32% Mild: 57.7% Moderate: 6.1% Severe: 4.4% Lobular/portal inflammation:	Elevated ferritin: approximately 35% of the included patients Cut-offs: 150 ng/ml in females and 300 ng/ml in males	χ^2^ test: Ferritin and steatosis stage: *p* < 0.01 Ferritin and inflammation stage: *p* < 0.01 Ferritin and fibrosis stage: *p* < 0.01 Ferritin and cirrhosis (compared with simple steatosis and steatosis + inflammation/fibrosis): *p* < 0.01	When data were processed with the multivariate analysis, ferritin was not found to be an independent predictor of hepatic lesions.	Gender, age, BMI, insulin, OGTT, cholesterol, triglycerides.
								Absent: 20% Mild: 64.9% Moderate: 6.6% Severe: 8.5% Pericellular fibrosis: Absent: 37% Mild: 50.7% Moderate: 9.3% Severe: 3.0%				
Manousou, 2011 (35)	Cross-sectional study	UK	Adult NAFLD patients	111 (71 + 40)	54 ± 14	Liver biopsy	Liver biopsy Non-NASH: either NAFLD or those cases that were thought to be borderline NASH: having inflammation + /- fibrosis Fibrosis: Group 1 (none or mild fibrosis): patients with fibrosis stages of 0, 1 and 1A according to Kleiner et al. (20) Group 2 (moderate fibrosis to cirrhosis): patients with fibrosis stages of 1B, 1C, 2, 3 and 4 according to Kleiner et al. (20) Steatosis and fibrosis were assessed according to Kleiner et al. (20) Lobular inflammation: 1: 0 <2 foci per ×200 field 2: 2–4 foci per ×200 field 3: >4 foci per ×200 field Portal inflammation: 0: none to minimal 1: greater than minimal	Non-NASH: 47 (42.3) NASH: 64 (57.7) Fibrosis: F0: 41.7% F1: 25% F2: 13% F3: 9.3% F4: 11.1% Lobular inflammation: 0: 28.7% 1: 50% 2: 20.4% 3: 0.9% Portal inflammation: 0: 49.1% 1: 50.9%	228 ± 100 Abnormal ferritin: 27 (24.5%) patients ULN: 150 ng/ml for females and 340 ng/ml for males	*T*-test: Ferritin and the occurrence of NASH: *p* < 0.001 Ferritin between two fibrosis groups: *p* < 0.001	Multivariate logistic regression analysis: Ferritin and the occurrence of NASH: 1.034 (1.003–1.161), *p* = 0.05 Ferritin and fibrosis stage (excluding 11 cirrhosis patients): 1.016 (1.007–1.024), *p* < 0.001 Ferritin and portal inflammation: 1.019 (1.008–1.022), *p* = 0.035 Ferritin and lobular inflammation: 1.056 (1.015–1.099), *p* = 0.007 Serum ferritin at a cut-off value of 240 ng/ml and above was significantly associated with lobular inflammation (*p* = 0.009) and portal inflammation (*p* = 0.043).	Ferritin and the occurrence of NASH: adjusted for BMI, DM, AST; Ferritin and fibrosis stage: adjusted for BMI; Ferritin and portal inflammation stage: adjusted for DM; Ferritin and lobular inflammation stage: adjusted for BMI and DM.
Moon, 2006 (36)	Cross-sectional study	South Korea	Adult NAFLD patients	39 (33 + 6)	34.5 ± 13.7	Liver biopsy	Liver biopsy Inflammation and fibrosis were staged according to Brunt et al. (22)	Inflammation: Grade 0: 5 (12.8) Grade 1: 16 (41.0) Grade 2: 16 (41.0) Grade 3: 2 (5.1)	250.5 ± 243.9	Correlation analysis: Ferritin and inflammation stage: *r* = 0.518, *p* = 0.001; Ferritin and fibrosis stage: *r* = 0.460, *p* = 0.005	Multivariate logistic regression analysis: Inflammation Grade 0 and 1	Age and BMI
								Fibrosis: Stage 0: 7 (17.9) Stage 1: 15 (38.5) Stage 2: 15 (38.5) Stage 3: 2 (5.1) Stage 4: 0 (0)		Univariate analysis: Inflammation Grade 0 and 1 were categorized as mild, and Grade 2 and 3 were categorized as moderate to conduct analysis; fibrosis Grade 0 and 1 were categorized as mild, and Grade 2, 3 and 4 were categorized as moderate to conduct analysis Ferritin and inflammation progression: *p* = 0.008 Ferritin and fibrosis progression: *p* = 0.035	were categorized as mild, and Grade 2 and 3 were categorized as moderate to conduct analysis; fibrosis Grade 0 and 1 were categorized as mild, and Grade 2, 3 and 4 were categorized as moderate to conduct analysis Ferritin and inflammation progression: regression coefficient 0.146, *p* = 0.303 Ferritin and fibrosis progression: regression coefficient 0.024, *p* = 0.531	
Mousavi, 2018 (37)	Cross-sectional study	Iran	Adult NAFLD patients	30 (17 + 13)	37.93 ± 12.5	Liver biopsy	Liver biopsy Staging and grading were performed according to the Brunt et al. (22) scoring	No steatohepatitis: 5 (16.7) Mild steatohepatitis: 19 (63.3) Moderate steatohepatitis: 4 (13.3) Severe steatohepatitis: 2 (6.7) Fibrosis: F0: 13 (43.3) F1: 3 (10) F2: 8 (26.7) F3: 4 (13.3) F4: 2 (6.7)	200.8 ± 200.6 Ferritin levels above 200: 11 (36.7%) patients Cut-off: 200ng/ml	Ferritin between patients without and with steatohepatitis: *p* > 0.05 (*t*-test) Ferritin among three grades of steatohepatitis: *p* = 0.559 (ANOVA test) Ferritin and fibrosis stage: *p* = 0.228 (correlation analysis)	N/A	N/A
Parikh, 2015 (38)	Case-control study	India	Group 1: healthy adults (control group) Group 2: adult NAFLD patients	105 (77 + 28) Group 1: 50 (37 + 13) Group 2: 55 (40 + 15)	Group 1: 41.6 ± 13.89 Group 2: 42.37 ± 3.2	Liver biopsy	Liver biopsy NAFL: steatosis with or without inflammation; NASH: steatosis with either ballooning or Mallory Denk bodies, bridging fibrosis or cirrhosis	NAFL: 35 NASH: 20 (all with fibrosis/cirrhosis)	Group 1: 35.2 ± 18.5 Group 2: 51.2 ± 9.4	Ferritin between NAFL and NASH patients: *p* < 0.05 (*t*-test) Ferritin (cut off 48 ng/ml) between Brunt fibrosis stages (0–2 and 3/4): *p* < 0.05 (χ^2^ test)	N/A	N/A
Ryan, 2018 (39)	Case-control study	UK	Group 1: healthy adults (control group) Group 2: adult NAFLD patients Group 3: adult HBV or HCV infected patients Group 4: adult NAFLD patients (validation group)	505 (398 + 107) Group 1: 20 (8 + 12) Group 2: 51 (32 + 19) Group 3: 30 (25 + 5) Group 4 :404 (333 + 71)	Group 1: 58 ± 10 Group 2: 55 ± 12.7 Group 3: 50 ± 12 Group 4: 49 ± 12	Liver biopsy	Liver biopsy and MRI Liver biopsy: Fibrosis and steatosis stage was determined as outlined by Brunt et al. (22) NASH: NAS score ≥5 MRI: Steatosis was determined by hepatic lipid content (HLC)	MRI HLC in Group 2: 15.2 ±12% Fibrosis in Group 2: Mild: 17 (33.3%) Moderate: 16 (31.4%) Severe: 18 (35.3%) NASH in Group 4: 171 (49.3%) patients	Group 2: 137 (rage 1,944) Group 4: 2.5 ± 0.5 log10 ng/ml Hyperferritinaemia: 7 patients in Group 2	Group 2: Correlation analysis: Ferritin and MRI-proven steatosis stage (HLC value): *r* = 0.57, *p* < 0.0001 Ferritin and histological steatosis: *r* = 0.5, *p* = 0.0002 Ferritin and histological inflammatory grade: *r* = 0.07, *p* = 0.62 ANOVA test: Ferritin and fibrosis stages (F0/1, F2, F3, F4): *p* = 0.007 Ferritin and the occurrence of NASH: *p* = 0.12 Group 4: Ferritin and histological grade of steatosis: *r* = 0.05, *p* = 0.33 Ferritin and lobular necroinflammation: *r* = 0.11, *p* = 0.021 Ferritin and hepatocellular ballooning: *r* = 0.02, *p* = 0.71 Ferritin increased significantly from F0/1 stage to F3 (*p* = 0.013), and then decreased (*p* = 0.048) in cirrhosis Linear regression analysis: Ferritin (log10 ng/ml) and steatosis: 0.03 (0.03), *p* = 0.33 Ferritin (log10 ng/ml) and inflammation: β = 0.07 (0.03), *p* = 0.02 Ferritin (log10 ng/ml) and fibrosis: β = 0.03 (0.02), *p* = 0.22	Group 2: Multiple logistic regression analysis: Ferritin was an independent predictor of significant (F2) compared with early (F0/1) fibrosis stage: OR (95% CI): 1.01 (1.00–1.014), *p* = 0.048 Group 4: Linear regression analysis: Ferritin (log10 ng/ml) and inflammation: β = 0.08 (0.03), *p* = 0.002	Group 2: adjusted for age, gender, weight, ALT adiponectin, HOMA-IR, propeptide of Type III Procollagen (P3NP), hepcidin, and MR liver T2; Group 4: adjusted for age, sex, type 2 diabetes, and alcohol.
Seyedian, 2017 (40)	Cross-sectional study	Iran	Adult NAFLD patients	284 (202 + 82)	Not reported	Not reported	Liver elastography Liver stiffness: Advanced stiffness: F3: >8.7 KPa F4: >10.3 KPa Liver cirrhosis: 11.5KPa	Mild liver stiffness: 226 Advanced liver stiffness: 58	High ferritin: 46 (16.2) patients Low ferritin: 238 (83.8) patients Cut-offs: 135 ng/ml in female and 225 ng/ml in male Mild liver stiffness group: 132 ± 101.6 Advanced liver stiffness group: 222.8 ± 194.4	Ferritin and liver stiffness level: *p* < 0.001 (*t*-test) Ferritin level and liver stiffness level: *p* < 0.001 (χ^2^ test)	N/A	N/A
Uysal, 2011 (41)	Case-control study	Turkey	Group 1: healthy adults (control group) Group 2: Adult NASH patients	88 (47 + 41) Group 1: 28 (10 + 18) Group 2: 60 (37 + 23)	Group 1: 48 ± 11 Group 2: 48 ± 14	Liver ultrasound	Liver ultrasound Patients with NASH was divided into three subgroups according to Saadeh et al. (42) minimal, moderate and marked steatosis subgroup parallel to the increase in echogenicity	Minimal steatosis: 17 Moderate steatosis: 20 Marked steatosis: 23	Group 1: 26.72 ± 11.26 Group 2: 117.54 ± 62.88 Minimal steatosis: 84.4 ± 39.1 Moderate steatosis: 105.5 ± 58.2 Marked steatosis: 152.4 ± 65.6	Kruskal–Wallis test or Mann–Whitney U test: Ferritin between minimal and marked steatosis: *p* < 0.05 Ferritin between moderate and marked steatosis: *p* < 0.05	N/A	N/A
Yao, 2019 (43)	Cross-sectional study	China	Non-obese general population taking their annual health examination, including NAFLD patients	1,020 (701 + 319) NAFLD patients: 148 (95 + 53)	43.4 ± 7.4 NAFLD patients: 23.4 ± 1.1	Liver ultrasound	Laboratory testing and age FIB-4 score (consists of age, AST, PLT, ALT level): Lower risk of fibrosis: FIB-4 <1.3 Advanced fibrosis: FIB-4 ≥ 1.3	Not reported	NAFLD patients: 276.7 (34.5–786.6) Low risk fibrosis group: 239.0 (34.5–326.6) Advanced fibrosis group: 308.8 (64.1–786.6)	Univariate logistic regression analysis: Ferritin and fibrosis stage: OR (95% CI): 2.760 (2.169–3.342), *p* < 0.001	Multivariate logistic regression analysis: The ORs (95% CI) and *p*-values of the associations of ferritin and fibrosis stage are as follows: When adjusted for age, gender, and BMI: 1.898 (1.163–2.621), *p* < 0.001 When adjusted for age, gender, BMI, uric acid (UA), and hypersensitive-CRP (hsCRP): 1.720 (1.149–2.302), *p* < 0.001 When adjusted for age, gender, BMI, UA, hsCRP, and hemoglobin: 1.401 (1.091–1.714), *p* = 0.02	Age, gender, BMI, UA, hsCRP, and hemoglobin
Yoneda, 2010 (44)	Case-control study	Japan	Group 1: healthy adults (control group) Group 2: adult NAFLD patients	106 (Sex not reported) Group 1: 20 Group 2: 86	Not reported	Liver biopsy	Liver biopsy Criteria for the diagnosis of NAFL and NASH were not reported	NAFL patients: 24 NASH patients: 62	NAFL: 164.9 ± 95.5 NASH: 278.6 ± 156.3	*T*-test: Ferritin and NAFL/NASH: *p* = 0.0060	N/A	N/A
Bugianesi, 2004 (45)	Cross-sectional study	Italy	Adult NAFLD patients	167 (Sex not reported)	41 ± 11	Liver ultrasound	Liver ultrasound Steatosis, necroinflammation and fibrosis were graded according to Brunt et al. (22) with minor modifications NASH was diagnosed based on the presence of fibrosis (grade 1 or higher) or necroinflammation (grade 2 or higher)	Steatosis: 1: 88 (52.7) 2: 47 (28.1) 3: 32 (19.2) Necroinflammation: 0: 16 (9.6) 1: 74 (44.3) 2: 55 (32.9) 3: 22 (13.2) Fibrosis: 0: 63 (37.7) 1: 38 (22.8) 2: 30 (18.0) 3: 27 (16.2) 4: 9 (5.4)	239 ± 235 Cut-off: 350 ng/ml	Univariate logistic regression analysis: Ferritin between mild fibrosis (stage 1–2) and no fibrosis (stage 0): OR (95% CI): 1.32 (1.06–1.67), *p* = 0.017 Ferritin between severe fibrosis (stage 3–4) and no fibrosis (stage 0): OR (95% CI):1.49 (1.18–1.88), *p* = 0.001 Linear regression analysis: Ferritin and steatosis stage: *r* = 0.309, *p* < 0.0001 Ferritin and fibrosis stage: *r* = 0.311, *p* < 0.0001 Ferritin and inflammation stage: *r* = 0.041, *p* = 0.601	Multivariate logistic regression analysis: Ferritin between mild fibrosis (stage 1–2) and no fibrosis (stage 0): OR (95% CI): 1.52 (1.08–2.13), *p* = 0.016 Ferritin between severe fibrosis (stage 3–4) and no fibrosis (stage 0): OR (95% CI): 1.69 (1.18–2.43), *p* = 0.0045	Age, sex, and BMI
Shimada, 2002 (46)	Cross-sectional study	Japan	Adult NASH patients	81 (40 + 41)	Median age: 54 (range 21–82)	Liver biopsy	liver biopsy Steatosis and fibrosis were graded according to Brunt et al. (22) Fibrosis was also graded as mild (F0–2) or severe (F3–4)	Mild fibrosis: 58 Severe fibrosis: 23 Severity of fibrosis: F0: 8 (10) F1: 29 (36) F2: 21 (26) F3: 6 (7) F4: 17 (21)	120 (range 13–520) Cut-off: 200ng/ml F0–2 fibrosis: 140 (27–520) F3–4 fibrosis: 67 (13–250)	Mann-Whitney test: Ferritin between mild and severe fibrosis: *p* = 0.0101	N/A	N/A
Angulo, 1999 (47)	Cross-sectional study	US	Adult NASH patients	144 (47 + 97)	50.5 (range 11–77)	Liver biopsy	Liver biopsy Degree of fibrosis: 0 = none, normal connective tissue 1 = mild, foci of pericellular fibrosis in zone 3 2 = moderate, perivenular or pericellular fibrosis confined to zone 3 and 2 regions, with or without portal/periportal fibrosis 3 = severe, bridging or septal fibrosis 4 = cirrhosis The level of fatty infiltration: 1 = mild (10%−30% of hepatocytes affected) 2 = moderate (30%−70% of hepatocytes affected) 3 = severe (>70% of hepatocytes affected)	Degree of fibrosis: 0: 37 (26) 1: 53 (37) 2: 15 (10) 3: 14 (10) 4: 25 (17) Degree of steatosis: 1: 40 (28) 2: 83 (58) 3: 21 (15)	221 (6–1,639) Elevated serum ferritin (>200): 77 (53%) patients Cut-off: 200ng/ml Degree of fibrosis: 0: 229 (24–1,520) 1–2: 242 (6–1,639) 3–4: 194 (11–1,000) Degree of fat infiltration: 0: 197 (24–1,040) 1–2: 246 (11–1,639) 3–4: 149 (6–1,000)	Kruskal-Wallis and Mann-Whitney tests: Ferritin and degree of fibrosis: *p* = 0.4 Ferritin and degree of fat infiltration: *p* = 0.1 χ^2^ test: Elevated ferritin and degree of fibrosis: *p* = 0.5 Elevated ferritin and degree of fat infiltration: *p* = 0.7	N/A	N/A
Koruk, 2003 (48)	Case-control study	Turkey	Group 1: healthy adults (control group) Group 2: adult NASH patients	34 (24 + 10) Group 1: 16 (11 + 5) Group 2: 18 (13 + 5)	Group 1: 40 ± 10.3 Group 2: 44 ± 7.1	Liver biopsy	Liver biopsy Inflammation, fibrosis and steatosis were graded according to Brunt et al. (22)	Steatosis: 1: 6 (33.3) 2: 10 (55.5) 3: 2 (11.1) Inflammation: Minimal: 7 (38.8) Mild: 8 (44.4) Moderate: 3 (16.6) Severe: 0 Fibrosis: 0: 8 (44.4) 1: 7 (38.8) 2: 3 (16.6) 3: 0 4: 0	Group 1: 81.87 ± 54.70 Group 2: 173.11 ± 91.04	There was no relationship between the serum concentrations of ferritin and the degree of hepatic steatosis, inflammation, and liver fibrosis in patients with NASH	N/A	N/A
Qu, 2021 (49)	Cross-sectional study	China	Adult NAFLD patients	167 (126 + 41)	S0: 38.45 ± 9.34 S1: 41.97 ± 12.55 S2: 43.55 ± 12.45 S3: 38.09 ± 11.22	Liver biopsy	Liver biopsy Inflammation, fibrosis and steatosis were graded according to Brunt et al. (22)	Steatosis: S0: 58 S1: 53 S2: 29 S3: 27	S0 patients: 206.20 ± 169.83 S1 patients: 286.65 ± 150.80 S2 patients: 326.55 ± 214.71 S3 patients: 391.50 ± 184.93	ANOVA test: Ferritin and steatosis stage: *p* = 0.006 Ferritin and inflammation stage: *p* = 0.470 Ferritin and fibrosis stage: *p* = 0.238	N/A	N/A
Trasolini, 2022 (50)	Retrospective cohort study	Canada	Adult NAFLD patients	224 (112 + 112)	52 (range 43–60)	Transient elastography	Transient elastography Low likelihood of fibrosis (>F1): <8.0 kPa High likelihood of significant fibrosis (F3–F4): >8.7 kPa	No fibrosis (<8.0 kPa): 185 Fibrosis (≥8.0 kPa): 39 Significant fibrosis (≥8.7 kPa): 32	145 (range 62–311) No fibrosis: 135 (range 60–304) Fibrosis: 161 (range 82–365)	Kruskal-Wallis test: Ferritin (cut-off of 300 ng/ml) and fibrosis stages: *p* = 0.099 Ferritin (cut-off of 450 ng/ml) and fibrosis stages: *p* = 0.12	N/A	N/A
Wang, 2022 (51)	Cross-sectional study	China	Adult NAFLD patients	136 (90 + 46)	41.00 (range 33.00–57.75)	Liver biopsy	Liver biopsy Steatosis, ballooning, and inflammation were graded according to the Steatosis-Activity-Fibrosis scoring system. (52) Steatosis: S0: less than 5% S1: 5–33% S2: 34–66% S3: more than 67% Hepatocellular ballooning: 0 point: normal cuboidal hepatocytes with pink eosinophilic cytoplasm 1 point: the presence of clusters of rounded hepatocytes with pale cytoplasm usually reticulated and quite similar size to that of normal hepatocyte although the shape is different 2 points: the presence of at least one enlarged hepatocyte with the size of 2-fold or more than that of normal cells based on features of 1 point Lobular inflammation:	Steatosis S1: 39 (28.7) S2: 55 (40.4) S3: 42 (30.9) Steatosis 2–3: 97 (71.3) Steatosis 3: 42 (30.9) Inflammation activity 1 point: 2 (1.5) 2 points: 24 (17.6) 3 points: 40 (29.4) 4 points: 70 (51.5) Inflammation activity 3–4: 110 (80.9) Inflammation activity 4: 70 (51.5) Fibrosis F1: 18 (13.2) F2: 71 (52.2) F3: 32 (23.5) F4: 15 (11.0) Fibrosis 2–4: 118 (86.8) Fibrosis 3–4: 47 (34.6) Fibrosis 4: 15 (11.0)	Normal ferritin: 79 (58.1%) patients Elevated ferritin: 57 (41.9%) patients Cut-offs: 336.2ng/ml in males and 306.8 ng/ml in females	χ^2^ test: Ferritin between Steatosis 2–3 and Steatosis 1: *p* = 0.040 Ferritin between Steatosis 3 and Steatosis 1–2: *p* = 0.599 Ferritin between inflammation activity 3–4 and inflammation activity 1–2: *p* = 0.085 Ferritin between inflammation activity 4 and inflammation activity 1–3: *p* = 0.021 Ferritin between Fibrosis 2–4 and Fibrosis 1: *p* = 0.069 Ferritin between Fibrosis 3–4 and Fibrosis 1–2: *p* = 0.116 Ferritin between Fibrosis 4 and Fibrosis 1–3: *p* = 0.692	N/A	N/A
							0 point: 0 inflammatory cell foci per 20x 1 point: ≤ 2 inflammatory cells foci per 20x 2 points: >2 inflammatory cells foci per 20x Inflammation activity score: the sum of lobular inflammation and hepatocellular ballooning. The severity of fibrosis was graded according to Kleiner et al. (20)					
Yang, 2022 (53)	Cross-sectional study	US	Adult NAFLD patients	1,604 (856 + 748)	52.73 ± 16.26	Vibration controlled and transient elastography	Vibration controlled and transient elastography Steatosis: Severe steatosis (S3): CAP ≥ 302 dB/m Fibrosis: Significant fibrosis (≥F2): LSM ≥8 kPa Advanced fibrosis (≥F3): LSM ≥ 9.7 kPa Cirrhosis (F4): LSM ≥ 13.6 kPa	CAP: 322.20 ± 36.09 dB/m LSM: 6.37 ± 4.84 kPa	166.41 ± 161.36	Univariate linear regression analysis: Dependent variable: serum ferritin levels Severe steatosis (S3) CAP <302: Reference CAP ≥ 302: 9.1 (−14.1 to 32.2), *p* = 0.443 Significant fibrosis (≥F2) LSM <8.0: Reference LSM ≥ 8.0: 95.4 (59.4–131.4), *p* < 0.001 Advanced fibrosis (≥F3) LSM <9.7: Reference LSM ≥ 9.7: 74.2 (44.8–103.7), *p* < 0.001 Cirrhosis (F4) LSM <13.6: Reference LSM ≥ 13.6: 147.9 (93.2–202.6), *p* < 0.001	Multivariate linear regression analysis: Dependent variable: serum ferritin levels Model 1: Severe steatosis (S3) CAP <302: Reference; CAP ≥ 302: −1.1 (−23.6 to 21.5), *p* = 0.925 Significant fibrosis (≥F2) LSM <8.0: Reference; LSM ≥ 8.0: 84.3 (49.3–119.4), *p* < 0.001 Advanced fibrosis (≥F3) LSM <9.7: Reference; LSM ≥ 9.7: 65.5 (36.8–94.1), *p* < 0.001 Cirrhosis (F4) LSM <13.6: Reference; LSM ≥ 13.6: 141.6 (88.5–194.6), *p* < 0.001 Model 2: Severe steatosis (S3) CAP <302: Reference; CAP ≥ 302: −7.3 (−29.0 to 14.4), *p* = 0.508 Significant fibrosis (≥F2) LSM <8.0: Reference; LSM ≥ 8.0: 5.9 (−29.5 to 41.2), *p* = 0.745 Advanced fibrosis (≥F3)	Model 1: age, gender, and race; Model 2: age, gender, race, BMI, diabetes, waist circumference, HDL-cholesterol, glycohemoglobin, AST, ALT, GGT, serum albumin, serum creatinine, and uric acid.
											LSM <9.7: Reference; LSM ≥ 9.7: −0.7 (−29.8 to 28.4), *p* = 0.960 Cirrhosis (F4) LSM <13.6: Reference; LSM ≥ 13.6: 38.9 (−15.2 to 93.0), *p* = 0.159	
Yu, 2022 (54)	Retrospective cohort study	US	Adult NAFLD patients	18,569 (6,990 + 11,579)	At baseline Group 1: 66.1 ± 10.8 Group 2: 59.9 ± 12.0	Not reported	Not reported	Group 1 (incident HCC during follow-up): 244 Group 2 (free of HCC during follow-up): 18,325	Median (range:5%−95%) Group 1: 83 (8–981) Group 2: 100 (9–700) Cut-offs: 200 ng/ml in females and 300 ng/ml in males	Wilcoxon rank sum test: Ferritin between Group 1 and Group 2: *p* = 0.445	Cox proportional hazard regression: Independent variable: serum ferritin level Dependent variable: incident HCC Normal ferritin: Reference Low ferritin (<30 ng/ml in males or <10 ng/ml in females): 1.38 (0.91, 2.09), *p* = 0.127 High ferritin: (>200 ng/ml in females and >300 ng/ml in males): 1.03 (0.75, 1.42), *p* = 0.868 *p* for trend: 0.368	Age, race, BMI, history of type 2 diabetes, cigarette smoking status

SD, standard deviation; NAFLD, non-alcoholic fatty liver disease; OR, odds ratio; CI, confidence interval; NASH, non-alcoholic steatohepatitis; ULN, upper limit of normal; BMI, body mass index; ALT, Alanine aminotransferase; NAFL, non-alcoholic fatty liver; NAS, NAFLD activity score; HOMA-IR, homeostatic model assessment insulin resistance index; OGTT, oral glucose tolerance test; DM, diabetes mellitus; AST, aspartate aminotransferase; HR, hazards ratio; CAP, controlled attenuation parameter; LSM, liver stiffness measurement; GGT, gamma glutamyl transpeptidase; HDL, high density lipoprotein; HCC, hepatocellular carcinoma.

Most studies utilized liver biopsy for NAFLD diagnosis and grading. The studies of Brunt et al. (22) and Kleiner et al. (20) were often referred to as the criteria for grading NAFLD progression, e.g., the grading of steatosis, inflammation and fibrosis stages. As the primary outcome of interest, the association of ferritin and various stages of NAFLD was proven by multivariate statistical analysis in 15 of the included studies, mostly adjusted for age, sex, BMI and other medical history variables. However, the other 17 studies only conducted univariate statistical analysis.

### Serum ferritin level and hepatic steatosis stages

Altogether, 15 studies investigated the association of serum ferritin level and hepatic steatosis stages in NAFLD patients (25, 26, 28–30, 32–34, 39, 41, 45, 48, 49, 51, 53). Among the 15 studies, nine studies consistently reported that NAFLD patients with a higher serum ferritin level were more likely to have an advanced steatosis stage (25, 28–30, 33, 34, 41, 45, 49), usually analyzed by correlation analysis. An Indian study reported significant associations in both females and males (25). Three studies did not find any significant association between serum ferritin level and steatosis stage (32, 48, 53). The other three studies reported inconsistent associations: one Egyptian study identified ferritin as a predictor for steatosis among NAFLD patients with hepatic fibrosis, but the association was not significant among patients without fibrosis (26); one study from the UK reported ferritin to be a predictor in one group of NAFLD patients, while it was not significantly related to steatosis progression in another group of NAFLD patients (39); the other study from China revealed that ferritin could distinguish Stage 2 or 3 steatosis from Stage 1, but not Stage 3 from Stage 1 or 2 (51). Almost all of the above results were tested by univariate statistical analysis without further exploration *via* multivariate analysis, except from two studies – one study reporting the predictive role of serum ferritin for steatosis progression that became non-significant in the multivariate analysis (34), and the other showed consistent non-significant associations in both univariate and multivariate analysis (53).

### Serum ferritin level and the occurrence of steatohepatitis

The association of serum ferritin level and the occurrence of steatohepatitis among NAFLD patients were investigated in 11 studies (19, 21, 23, 27, 28, 33, 35, 37–39, 44). Nine of the 11 studies compared the serum ferritin level in NAFL patients with it in NASH patients, among which five studies identified it to be a predictor for the occurrence of NASH (23, 27, 28, 35, 38, 44) yet two studies showed non-significant associations (21, 39). Data in these studies were usually analyzed by ANOVA test or *t*-test. Only three studies further included ferritin into a multivariate model and their result remained the same as it was in the univariate analysis (21, 27) except that in one study, the significant difference (*p* < 0.001) became borderline (*p* = 0.05) (35). Interestingly, one Italian study set 160 and 380 ng/ml as ferritin cut-offs and found both of them were predictive for the occurrence of NASH, with the cut-off of 380 ng/ml having a higher odds ratio in both univariate and multiple logistic regression analyses (27).

An international study and a study from the US compared ferritin levels among patients with different NASH categories – no NASH, suspicious or borderline NASH and definitive NASH, and found a significant difference among the three groups of patients *viaχ*^2^ test, but no further analysis was conducted to identify the trend of serum ferritin level in NASH progression (19, 33). Another Iranian study had similar results; there was a difference of serum ferritin levels among patients with mild, moderate and severe steatohepatitis, but no further comparison was made (37).

Five studies analyzed the accuracy of ferritin level for diagnosing NASH by conducting receiver operating characteristic (ROC) curve analysis (19, 28, 35, 38, 44) and reported inconsistent results (Table 2). Two suggested that ferritin had poor accuracy (19, 28), but three demonstrated the opposite end (35, 38, 44).

**Table 2:** Summary of receiver operating characteristic (ROC) curve analysis of included studies.

**Study (first author, year)**	**Ferritin cut-off value (ng/ml)**	**Diagnostic aim**	**Sensitivity (%), Specificity (%)**	**AUROC (95% CI)**	***p*-Value**
Angulo, 2014 (19)	>ULN (200 ng/ml in females and	Fibrosis stage 1–4 vs. stage 0	37, 76	0.57 (0.53–0.60)	Not reported
	300 ng/ml in males)	Fibrosis stage 2–4 vs. stage 0–1	39, 72	0.55 (0.52–0.59)	Not reported
		Fibrosis stage 3–4 vs. stage 0–2	41, 70	0.55 (0.51–0.59)	Not reported
	>1.5 ULN (300 ng/ml in females	Fibrosis stage 1–4 vs. stage 0	22, 89	0.55 (0.52–0.59)	Not reported
	and 450 ng/ml in males)	Fibrosis stage 2–4 vs. stage 0–1	25, 86	0.55 (0.52–0.59)	Not reported
		Fibrosis stage 3–4 vs. stage 0–2	27, 84	0.56 (0.52–0.60)	Not reported
	>2.0 ULN (400 ng/ml in females	Fibrosis stage 1–4 vs. stage 0	13, 95	0.54 (0.50–0.58)	Not reported
	and 600 ng/ml in males)	Fibrosis stage 2–4 vs. stage 0–1	14, 93	0.53 (0.50–0.57)	Not reported
		Fibrosis stage 3–4 vs. stage 0–2	16, 92	0.54 (0.50–0.58)	Not reported
	>ULN (200 ng/ml in females and 300 ng/ml in males)	The occurrence of NASH	Not reported	0.58 (0.54–0.61)	Not reported
El Nakeeb, 2017 (26)	≥51.95 ng/ml	The occurrence of fibrosis	65.4, 40	Not reported	Not reported
Hanafy, 2019 (30)	>321 ng/ml	Fibrosis stage 3–4 vs. stage 0–2	95.8, 90	0.809 (0.77–0.85)	0.001
Manousou, 2011 (35)	>240 ng/ml	The occurrence of NASH	91, 70	0.82 (0.73–0.90)	Not reported
Parikh, 2015 (38)	≥48 ng/ml	Fibrosis stage 3–4 vs. stage 0–2	Not reported	0.779 (95% CI not reported)	Not reported
Seyedian, 2017 (40)	>255 ng/ml (in males)	Advanced liver stiffness vs. mild liver stiffness	90, specificity not reported	0.59 (0.489–0.697)	Non-significant
	>135 ng/ml (in females)		Not reported	0.79 (0.663–0.917)	Significant
	<72.5 ng/ml (in males)	Excluding advanced liver stiffness	90, specificity not reported	Not reported	Not reported
	<65.5 ng/ml (in females)		93, specificity not reported	Not reported	Not reported
Yoneda, 2010 (44)	196 ng/ml	The occurrence of NASH	64.2, 76.5	0.732 (0.596–0.856)	0.005

AUROC, area under the ROC curve; CI, confidence interval; ULN, upper limit of normal; NASH, non-alcoholic steatohepatitis; NAFL, non-alcoholic fatty liver.

### Serum ferritin level and hepatic fibrosis stages

There are 25 studies exploring the association of serum ferritin level and fibrosis stages in NAFLD patients (19, 21, 23, 26–29, 31–37, 39, 40, 43, 45–51, 53).

#### Ferritin and hepatic fibrosis stages graded from F0–F4 using Brunt et al.'s standards

Most of these studies employed the Brunt et al. (22) standards to grade fibrosis stages from F0 (absence of fibrosis) to F4 (cirrhosis). In an international study, ferritin was reported to be significantly different in NAFLD patients with different stages of fibrosis *viaχ*^2^ test, and further identified that serum ferritin levels higher than the upper limit of normal (ULN, which was 200 ng/ml in females and 300 ng/ml in males), higher than 1.5 ULN and higher than 2 ULN were predictors of presence of fibrosis (F1–F4), severe fibrosis (F2–F4) and advanced fibrosis (F3/F4), respectively, through multiple logistic regressions (19). Similarly, a study from the US reported significant results in univariate analysis, and identified both 1.5 ULN and 2.5 ULN ferritin levels to be predictors of advanced fibrosis (33). A study from two European countries reported that when comparing with the ferritin level of patients with F0–F1 fibrosis, that of F2 patients were non-significantly different, while that of F3 and F4 patients were significantly different; however, multiple analysis showed non-significant results (21).

Different adjusting confounders would influence the associations. One study from the US reported that ferritin could distinguish significant fibrosis (F2–F4), advanced fibrosis (F3–F4) and cirrhosis (F4) from less severe fibrosis; the associations remained significant when age, gender and race were adjusted, yet became non-significant when more variables were included, e.g., BMI, medical history of diabetes, waist circumference, laboratory analysis results including alanine aminotransferase (ALT), etc. (53).

#### Different associations of ferritin and hepatic fibrosis stages

Ten studies simply reported patients with more advanced fibrosis were more likely to have a higher serum ferritin level (23, 29, 32, 34, 35, 38, 40, 43, 45, 46), tested by univariate analysis. Four of the 10 studies further included ferritin into multivariate analysis models, and three studies had results that remained the same (35, 43, 45) yet one study showed non-significant association (36). One study only had results from multivariate analysis and reported the significant association that a higher ferritin level predicts the presence of significant fibrosis (30). Another seven studies showed non-significant results in univariate analysis (28, 37, 47–51). Further, one study found non-significant association between ferritin and the occurrence of fibrosis in NAFLD patients (26). Additionally, one study reported inconsistent results from two different groups of NAFLD patients (39), and one study reported that ferritin was higher in patients with cirrhosis when comparing with patients with simple steatosis and steatosis plus inflammation or fibrosis (34).

Interestingly, when ferritin cut-offs were set as 160 ng/ml and 380 ng/ml, the differences of ferritin levels among patients with different fibrosis were non-significant for both two cut-offs in univariate analysis, but the association of ferritin level and fibrosis stages became significant when ferritin cut-off was 380 ng/ml in multiple logistic regression (27).

#### Ferritin and hepatic fibrosis progression in longitudinal study

A Japanese study using longitudinal data followed a group of NAFLD patients with F3 fibrosis at baseline, and categorized them into deterioration group (F4), no-change group (F3) and improvement group (F1/F2) according to their fibrosis stage at follow-up after 1–10 year(s) (31). This study showed that changes of ferritin levels in these patients were significantly different among the three groups, with significant differences in both between no-change group and deterioration group and between no-change group and improvement group (31).

#### Accuracy of ferritin for predicting hepatic fibrosis

Three studies further explored the accuracy of ferritin for predicting fibrosis stages (19, 30, 40). One study suggested it had poor accuracy among males yet had high accuracy among females (40), one study reported poor accuracy generally (19), and one study demonstrated it was a good predictor (30).

### Serum ferritin level and hepatic inflammation stages

Eleven studies explored the association serum ferritin level and inflammation stages among NAFLD patients (28, 29, 32–36, 39, 45, 48, 49). Ten of the 11 studies conducted univariate analysis: four studies demonstrated that NAFLD patients with a higher ferritin level were more likely to have more advanced hepatic inflammation (29, 33, 34, 36); five studies showed non-significant results (28, 32, 45, 48, 49); and one study reported inconsistent associations from two different groups of patients (39). Only three studies explored the association of ferritin and inflammation progression *via* multivariate analysis: one study identified ferritin as a predictor for more advanced portal and lobular inflammation status, with a significant cut-off value of 240 ng/ml (35); another study found non-significant associations of ferritin between patients with mild (Grade 0 and 1) and moderate (Grade 2 and 3) inflammation (36); the other study found significant association of ferritin (log 10 ng/ml) and inflammation stages by multiple linear regression analysis (39).

### Serum ferritin level and hepatic ballooning stages

The association of serum ferritin level and hepatic ballooning were investigated in five studies (28, 29, 32, 33, 39), all tested by univariate analysis. Three of them suggested that NALFD patients with higher ferritin were more likely to have a more advanced ballooning stage (28, 29, 33), and the other two reported non-significant results (32, 39).

Another one study from China combined inflammation and ballooning score as the inflammation activity score (1–4, the higher the more severe), and found that ferritin levels were different between patients with 4 points and 1–3 points, but not 3–4 points and 1–2 points (51).

### Serum ferritin level and integrated NAFLD progression including incident HCC and mortality

Three studies reported that serum ferritin level was positively correlated with NAFLD activity score (NAS) (28, 29, 32).

One study from the US explored the role of ferritin in predicting future incident hepatocellular carcinoma (HCC), with an average follow-up of 4.34 years. The authors reported non-significant associations both in univariate analysis and multivariate Cox proportional hazard regression analysis (54).

There is one study investigating the association of serum ferritin level and mortality (29). It suggested that following 15 years after liver biopsy, patients with elevated ferritin (>350 ng/ml in males and >150 ng/ml in females) showed a significant and gradually steeper increase in mortality compared with those with normal ferritin levels at biopsy; following 30 years after biopsy, the hazard ratio increased 9% faster per year in patients with elevated ferritin, and the significance remained when potential confounders were adjusted.

## Discussion

This systematic literature review identified 32 studies reporting the association between serum ferritin level or different ferritin categories and various stages of NAFLD, including the occurrence of NASH, hepatic steatosis stages, fibrosis stages, inflammation stages, ballooning stages, incident HCC and mortality. Most studies suggested that serum ferritin was a predictor for more advanced NAFLD and could relate to higher mortality. However, non-significant association was also reported by a few included studies. The accuracy of ferritin as a predictor for NAFLD progression was also reported inconsistently.

This study not only synthesized current evidence on the association of ferritin and NAFLD progressions, but also identified certain research gaps in this field. First, more than half of the included studies only employed univariate statistical analysis. Under these circumstances, the reliability of the association was not high due to the potential influences exerted by confounders such as age, sex, ATL levels, etc. Future studies should apply a rigorous study design. Second, although many studies employed a cohort design, only three of them used longitudinal data for analysis (29, 31, 54). Two of them investigated the association of ferritin and future incident HCC (54) and mortality (29), respectively; the other revealed the association of ferritin and changes of fibrosis stages (31). This calls for more studies to explore the predictive value of ferritin for NAFLD prognosis. Third, when categorizing the included studies according to the WHO regions, we found that none of the studies were from the African Region, indicating a research gap among African populations. Fourth, heterogeneity was high among the included studies and it prevented further data synthesis *via* meta-analysis. The included studies used different grading standards and various statistical analysis approaches. Future studies could apply consistent study design for better homogeneity to assist data synthesis on this topic. Fifth, many studies did not evaluate the diagnosis accuracy, specificity, or sensitivity of serum ferritin level, without which the predictive value of ferritin for evaluating various stages of NAFLD would not be clear. Sixth, many studies used the same ferritin cut-off values for all participants, thus failed to observe the potential sex differences in the associations of ferritin and NAFLD stages between the two populations, since in addition to the sex differences in NAFLD prevalence, there are also differences in ferritin cut-off values as a result of different iron status between females and males (55). Future studies are supposed to take sex differences into consideration.

Several previous reviews have narratively summarized existing evidence on this topic, which mostly elaborated the association of serum ferritin and the occurrence of NASH (11, 16, 56, 57) or fibrosis (16, 56, 57). One meta-analysis identified that ferritin was independently associated with NAFLD and NASH diagnosis (58). Our study included a broader body of evidence and added insights into the associations of ferritin and stages of steatosis, inflammation and ballooning, indicating the potential value of ferritin as a biomarker for clinical assessment of NAFLD progression and prognosis. The inconsistent associations reported by the included studies might result from inadequate sample sizes in some of the included studies. Gene variants or polymorphisms that are relevant to iron metabolism could influence serum ferritin levels in NAFLD patients, which might provide an explanation for the non-significant role of serum ferritin in predicting NAFLD progression in certain studies (21). In addition, previous studies recommended ferritin to be a component of non-invasive integrated scoring system for NAFLD assessment (59, 60), since the diagnosis accuracy improved under this circumstance (61).

Studies have explored the mechanisms of the elevated serum ferritin and how it is related to disease various disease stages in NAFLD patients. On the one hand, NAFLD disease progression process can upregulate serum ferritin: one mechanism is that the excessive ferritin is released by damaged hepatocytes and/or systematic inflammation; inflammation can also upregulate hepcidin levels and result in dysmetabolic iron overload syndrome (DIOS), consequently raising up serum ferritin level; the p.C282Y homozygote HFE mutation in NAFLD patients is related to elevated transferrin saturation, accompanied by abnormally higher ferritin (13); further, signals that mediate NASH pathogenesis (e.g., TNF-α, IL-1β) can elevate ferritin level (33). On the other hand, ferritin is involved in NASH pathogenesis by promoting apoptosis and inducing signaling cascades related to inflammation, oxidative stress, lipid transport, and fibrogenesis (33).

To the best of our knowledge, this is the first study that has comprehensively synthesized and reported the existing evidence on this topic, including articles published in three languages and from various countries, and shedding light on the diagnostic and predictive value of ferritin for NAFLD assessment. However, some limitations should be noted. First, meta-analysis was not conducted due to the heterogeneity of NAFLD grading standards and statistical analysis methods among the included studies. Moreover, the absence of necessary data in some included studies for conducting meta-analysis prevented us from further analysis. Second, five studies were not incorporated in this study since we did not have access to their full texts. This might result in a certain level of bias.

In conclusion, serum ferritin level could be considered to act as a non-invasive biomarker for NAFLD progression assessment owing to its associations with the occurrence of NASH, the stages of steatosis, inflammation, ballooning, fibrosis, general NAFLD progression and mortality. Nevertheless, further studies are still in need to confirm its predictive value since this study reported inconsistent associations based on the qualitative synthesis.

## Data availability statement

The original contributions presented in the study are included in the article/Supplementary material, further inquiries can be directed to the corresponding author.

## Author contributions

CY conceptualized the study, supervised the project, and was acting as the submission's guarantor. HW and RS searched the literature, extracted, and coded the data, completed the visualization, and interpreted the results. HW prepared the original draft with important contributions from RS. SY, XM, and CY commented on drafts, and provided edits and feedback. All authors had full access to all the data and have approved the final version of the paper.

## Funding

The work was supported by grants from the National Scientific and Technological Major Project of China (No. 2017ZX10105001).

## Conflict of interest

The authors declare that the research was conducted in the absence of any commercial or financial relationships that could be construed as a potential conflict of interest.

## Publisher's note

All claims expressed in this article are solely those of the authors and do not necessarily represent those of their affiliated organizations, or those of the publisher, the editors and the reviewers. Any product that may be evaluated in this article, or claim that may be made by its manufacturer, is not guaranteed or endorsed by the publisher.
