# Supplementary material for: Association between serum ferritin level and the various stages of non-alcoholic fatty liver disease: A systematic review
Source: Front Med (Lausanne). 2022 Aug 3;9:934989. doi: 10.3389/fmed.2022.934989 (PMC9381877; doi:10.3389/fmed.2022.934989)
Supplement: Supplementary file 1 [file Data_Sheet_1.docx]

Supplementary Material

**Supplementary Table 1. Search strategies in the three bibliographic databases**

| **Database** | **Access date** | **Search terms** | **Number of identified records** |
| --- | --- | --- | --- |
| MEDLINE | 10th July, 2022 | (("fat liver"[tiab] OR "liver steatosis"[tiab] OR "hepatic steatosis "[tiab] OR Steatohepatitis[tiab] OR "fatty liver"[tiab] OR "fatty liver"[mesh]) AND ("ferritin*"[tiab] OR "ferritins"[mesh]) AND ("1920/09/01"[EDAT] :"2022/6/30"[EDAT]) AND ("1920/09/01"[PDAT] : "2022/6/30"[PDAT])) AND ("loattrfull text"[sb] NOT (animals[mh] NOT (humans[mh] AND animals[mh]))) | 376 |
| EMBASE | 10th July, 2022 | (('fatty liver'/exp OR 'hepatic steatosis':ti,ab,kw OR hepatosteatosis:ti,ab,kw OR 'liver fatty infiltration':ti,ab,kw OR 'liver, fatty':ti,ab,kw OR 'liver steatosis':ti,ab,kw OR 'steatotic liver':ti,ab,kw OR 'steatosis, liver':ti,ab,kw OR 'fat liver':ti,ab,kw OR 'fatty liver':ti,ab,kw OR 'fatty liver disease':ti,ab,kw OR 'fatty liver infiltration':ti,ab,kw OR 'fatty liver syndrome':ti,ab,kw) AND ('ferritin'/exp OR ferritin*:ti,ab,kw)) NOT ('animals'/de NOT ('humans'/de AND 'animals'/de)) AND [1-1-1920]/sd NOT [6-30-2022]/sd | 1271 |
| Scopus | 10th July, 2022 | ( ( TITLE-ABS-KEY ( fatty AND liver ) OR TITLE-ABS-KEY ( hepatic AND steatosis ) OR TITLE-ABS-KEY ( hepatosteatosis ) OR TITLE-ABS-KEY ( liver AND fatty AND infiltration ) OR TITLE-ABS-KEY ( liver,fatty ) OR TITLE-ABS-KEY ( liver AND steatosis ) OR TITLE-ABS-KEY ( steatotic AND liver ) OR TITLE-ABS-KEY ( steatosis,liver ) OR TITLE-ABS-KEY ( fat AND liver ) OR TITLE-ABS-KEY ( fatty AND liver AND disease ) OR TITLE-ABS-KEY ( fatty AND liver AND infiltration ) OR TITLE-ABS-KEY ( fatty AND liver AND syndrome ) ) ) AND ( TITLE-ABS-KEY ( ferritin ) ) | 1023 |

**Supplementary Table 2. Reference list of the included articles (n=32)**

| **ID** | **Reference** |
| --- | --- |
| 1 | Angulo P, George J, Day CP, et al. Serum ferritin levels lack diagnostic accuracy for liver fibrosis in patients with nonalcoholic fatty liver disease. Clin Gastroenterol Hepatol 2014;12:1163-1169.e1. |
| 2 | Angulo P, Keach JC, Batts KP, et al. Independent predictors of liver fibrosis in patients with nonalcoholic steatohepatitis. Hepatology 1999;30:1356-1362. |
| 3 | Bugianesi E, Manzini P, D’Antico S, et al. Relative contribution of iron burden, HFE mutations, and insulin resistance to fibrosis in nonalcoholic fatty liver. Hepatology 2004;39:179-187. |
| 4 | Buzzetti E, Petta S, Manuguerra R, et al. Evaluating the association of serum ferritin and hepatic iron with disease severity in non-alcoholic fatty liver disease. Liver Int 2019;39:1325-1334. |
| 5 | Canbakan B, Senturk H, Tahan V, et al. Clinical, biochemical and histological correlations in a group of non-drinker subjects with non-alcoholic fatty liver disease. Acta Gastro-Enterol Belg 2007;70:277-284. |
| 6 | Chandok N, Minuk G, Wengiel M, et al. Serum ferritin levels do not predict the stage of underlying non-alcoholic fatty liver disease. J Gastrointest Liver Dis 2012;21:53-58. |
| 7 | Chaturvedi M, Pal K, Verma R, et al. Prevalance of non-alcoholic fatty liver disease in hypothyroid patients and its correlation with seruml ferritin levels. J Indian Acad Clin Med 2020;21:43-45. |
| 8 | El Nakeeb N, Saleh SA, Massoud YM, et al. Serum ferritin as a non-invasive marker in the prediction of hepatic fibrosis among Egyptian patients with non-alcoholic fatty liver disease. JGH Open 2017;1:112-119. |
| 9 | Fracanzani AL, Valenti L, Bugianesi E, et al. Risk of nonalcoholic steatohepatitis and fibrosis in patients with nonalcoholic fatty liver disease and low visceral adiposity. J Hepatol 2011;54:1244-1249. |
| 10 | Goh GB, Issa D, Lopez R, et al. The development of a non-invasive model to predict the presence of non-alcoholic steatohepatitis in patients with non-alcoholic fatty liver disease. J Gastroenterol Hepatol 2016;31:995-1000. |
| 11 | Hagström H, Nasr P, Bottai M, et al. Elevated serum ferritin is associated with increased mortality in non-alcoholic fatty liver disease after 16 years of follow-up. Liver Int 2016;36:1688-1695. |
| 12 | Hanafy AS, Seleem WM, El-kalla F, et al. Efficacy of a non-invasive model in predicting the cardiovascular morbidity and histological severity in non-alcoholic fatty liver disease. Diabetes Metab Syndr Clin Res Rev 2019;13:2272-2278. |
| 13 | Kawanaka M, Oka T, Urata N, et al. Clinical characteristics of non-alcoholic steatohepatitis (NASH) patients who progressed from F3 stage fibrosis to cirrhotic NASH. J Jpn Soc Gastroenterolog 2012;109:2042-2048. (Published in Japanese) |
| 14 | Kim YS, Jung ES, Hur W, et al. Noninvasive predictors of nonalcoholic steatohepatitis in Korean patients with histologically proven nonalcoholic fatty liver disease. Clin Mol Hepatol 2013;19:120-130. |
| 15 | Koruk M, Tayşi S, Savaş MC, et al. Serum levels of acute phase proteins in patients with nonalcoholic steatohepatitis. Turk J Gastroenterol 2003;14:12-17. |
| 16 | Kowdley KV, Belt P, Wilson LA, et al. Serum ferritin is an independent predictor of histologic severity and advanced fibrosis in patients with nonalcoholic fatty liver disease. Hepatology 2012;55:77-85. |
| 17 | Loguercio C, De Simone T, D’Auria MV, et al. Non-alcoholic fatty liver disease: A multicentre clinical study by the Italian Association for the Study of the Liver. Dig Liver Dis 2004;36:398-405. |
| 18 | Manousou P, Kalambokis G, Grillo F, et al. Serum ferritin is a discriminant marker for both fibrosis and inflammation in histologically proven non-alcoholic fatty liver disease patients. Liver Int 2011;31:730-739. |
| 19 | Moon JH, Park SH, Oh KC, et al. Association of hepatic iron deposition and serum iron indices with hepatic inflammation and fibrosis stage in nonalcoholic fatty liver disease. Korean J Gastroenterol 2006;47:432-439. (Published in Korean) |
| 20 | Mousavi SRM, Geramizadeh B, Anushiravani A, et al. Correlation between serum ferritin level and histopathological disease severity in non-alcoholic fatty liver disease. Middle East J Dig Dis 2018;10:90-95. |
| 21 | Parikh P, Patel J, Ingle M, et al. Serum ferritin levels predict histological severity in patients with nonalcoholic fatty liver disease in India. Indian J Gastroenterol 2015;34:200-208. |
| 22 | Ryan JD, Armitage AE, Cobbold JF, et al. Hepatic iron is the major determinant of serum ferritin in NAFLD patients. Liver Int 2018;38:164-173. |
| 23 | Seyedian SS, Hajiani E, Hashemi SJ, et al. Relationship between serum ferritin level and transient elastography findings among patients with nonalcoholic fatty liver disease. J Family Med Prim Care 2017;6:750-754. |
| 24 | Shimada M, Hashimoto E, Kaneda H, et al. Nonalcoholic steatohepatitis: risk factors for liver fibrosis. Hepatol Res 2002;24:429-438. |
| 25 | Uysal S, Armutcu F, Aydogan T, et al. Some inflammatory cytokine levels, iron metabolism and oxidan stress markers in subjects with nonalcoholic steatohepatitis. Clin Biochem 2011;44:1375-1379. |
| 26 | Yao J, Dai Y, Zhang J, et al. Association between serum ferritin level and nonalcoholic fatty liver disease in a non-obese Chinese population: A cross-sectional study. Clin Lab 2019;65. |
| 27 | Yoneda M, Nozaki Y, Endo H, et al. Serum ferritin is a clinical biomarker in Japanese patients with nonalcoholic steatohepatitis (NASH) independent of HFE gene mutation. Dig Dis Sci 2010;55:808-814. |
| 28 | Qu HJ, Wang L, Zhuang ZJ, et al. [Studying the correlation between ferritin and non-alcoholic fatty liver disease]. Zhonghua Gan Zang Bing Za Zhi 2021;29:1089-1094. |
| 29 | Trasolini R, Cox B, Galts C, et al. Elevated serum ferritin in non-alcoholic fatty liver disease is not predictive of fibrosis. Canadian Liver J 2022;5:152-159. |
| 30 | Wang Q, Zhu M, Li H, et al. Hyperferritinemia Correlates to Metabolic Dysregulation and Steatosis in Chinese Biopsy-Proven Nonalcoholic Fatty Liver Disease Patients. Diabetes Metab Syndr Obes 2022;15:1543-1552. |
| 31 | Yang N, Lu Y, Cao L, et al. The association between non-alcoholic fatty liver disease and serum ferritin levels in American adults. J Clin Lab Anal 2022;36:e24225. |
| 32 | Yu Y-C, Luu HN, Wang R, et al. Serum Biomarkers of Iron Status and Risk of Hepatocellular Carcinoma Development in Patients with Nonalcoholic Fatty Liver Disease. Cancer Epidemiol Biomarkers Prev 2022;31:230-235. |

**Supplementary Table 3. Quality assessment of the included studies**

| **Author, Year** | **1.Describe and justify clinical question** | **2.Describe patients included** | **3.Describe sampling** | **4.Describe experimental methodology** | **5.Describe statistical evaluation** | **6.Validation of results** | **7.Limitations are acknowledged** | **8.Contributions of each author is stated** |
| --- | --- | --- | --- | --- | --- | --- | --- | --- |
| Angulo, P., 2014 | Yes | Yes | Yes | Yes | Yes | Yes | No | Yes |
| Buzzetti, E., 2019 | Yes | Yes | Yes | Yes | Yes | Yes | Yes | No |
| Canbakan, B., 2007 | Yes | Yes | Yes | Yes | Yes | Yes | No | No |
| Chandok, N., 2012 | Yes | Yes | Yes | Yes | Yes | Yes | Yes | No |
| Chaturvedi, M., 2020 | Yes | Yes | Yes | Yes | No | Yes | No | No |
| El Nakeeb, N., 2017 | Yes | Yes | Yes | Yes | Yes | Yes | No | No |
| Fracanzani, A.L., 2011 | Yes | Yes | Yes | Yes | Yes | Yes | No | No |
| Goh, G.B., 2016 | Yes | Yes | Yes | Yes | Yes | Yes | Yes | No |
| Hagstrom, H., 2016 | Yes | Yes | Yes | Yes | Yes | Yes | Yes | Yes |
| Hanafy, A.S., 2019 | Yes | Yes | Yes | Yes | Yes | Yes | Yes | No |
| Kawanaka, M., 2012 | Yes | Yes | Yes | Yes | Yes | Yes | No | No |
| Kim, Y.S., 2013 | Yes | Yes | Yes | Yes | Yes | Yes | Yes | No |
| Kowdley, K.V., 2012 | Yes | Yes | Yes | Yes | Yes | Yes | Yes | No |
| Loguercio, C., 2004 | Yes | Yes | Yes | Yes | Yes | Yes | No | No |
| Manousou, P., 2011 | Yes | Yes | Yes | Yes | Yes | Yes | No | No |
| Moon, J.H., 2006 | Yes | Yes | Yes | Yes | Yes | Yes | No | No |
| Mousavi, S.R.M., 2018 | Yes | Yes | Yes | Yes | Yes | Yes | Yes | No |
| Parikh, P., 2015 | Yes | Yes | Yes | Yes | Yes | Yes | Yes | No |
| Ryan, J.D., 2018 | Yes | Yes | Yes | Yes | Yes | Yes | No | No |
| Seyedian, S.S., 2017 | Yes | Yes | Yes | Yes | No | Yes | No | No |
| Uysal, S., 2011 | Yes | Yes | Yes | Yes | Yes | Yes | No | No |
| Yao, J., 2019 | Yes | Yes | Yes | Yes | Yes | Yes | Yes | Yes |
| Yoneda, M., 2010 | Yes | Yes | Yes | Yes | Yes | Yes | No | Yes |
| Bugianesi, E., 2004 | Yes | Yes | Yes | Yes | Yes | Yes | No | No |
| Shimada, M., 2002 | Yes | Yes | Yes | Yes | Yes | Yes | No | No |
| Angulo, P., 1999 | Yes | Yes | Yes | Yes | Yes | Yes | No | No |
| Koruk, M., 2003 | Yes | Yes | Yes | Yes | Yes | Yes | No | No |
| Qu, H., 2021 | Yes | Yes | Yes | Yes | Yes | Yes | Yes | Yes |
| Trasolini, R., 2022 | Yes | Yes | Yes | Yes | Yes | Yes | No | Yes |
| Wang, Q., 2022 | Yes | Yes | Yes | Yes | Yes | Yes | Yes | Yes |
| Yang, N., 2022 | Yes | Yes | Yes | Yes | Yes | Yes | Yes | Yes |
| Yu, Y., 2022 | Yes | Yes | Yes | Yes | Yes | Yes | Yes | Yes |
